# The transgenic expression of the β-subunit of human chorionic gonadotropin influences the growth of implanted tumor cells

**DOI:** 10.18632/oncotarget.26158

**Published:** 2018-10-05

**Authors:** Poonam Singh, Moumita Sarkar, Usha Agrawal, Ilpo Huhtaniemi, Rahul Pal

**Affiliations:** ^1^ Immunoendocrinology Lab, National Institute of Immunology, Aruna Asaf Ali Marg, New Delhi, INDIA-110067; ^2^ National Institute of Pathology, Safdarjang Hospital Campus, Ansari Nagar West, New Delhi, INDIA-110029; ^3^ Department of Surgery & Cancer, Imperial College London, South Kensington Campus, London, UK SW7 2AZ

**Keywords:** human chorionic gonadotropin, gonadotropin-associated tumorigenesis, tumor-associated genes, tumor progression, prognosis

## Abstract

The beta subunit of human chorionic gonadotropin (βhCG) is secreted by various tumors, and its presence associated with poor prognosis. Though exogenous hCG elicits the synthesis of molecules associated with angiogenesis, invasion, immune suppression and chemoresistance from responsive tumor cells *in vitro*, the influence of cell-extrinsic βhCG on tumorigenesis *in vivo* has not been adequately explored. Female C57BL/6^−/−^ × FVB^βhCG/−^ F1 transgenic mice demonstrated ovarian hyperplasia and pituitary adenomas; transcripts of hCG-driven, tumor-associated molecules were heightened in the pituitary. Upon the implantation of Lewis Lung Carcinoma cells (murine lung tumor cells derived from C57BL/6 mice) in transgenic mice, tumor incidence and volume were enhanced, and increased transcription and expression of hCG-driven, tumor-associated molecules was observed in excised tumors. While treatment of these mice with Cabergoline (a potent dopamine receptor agonist) had no significant effects, ovariectomy resulted in a reduction in the lag phase, accompanied by an increase in tumor incidence and volume upon Lewis Lung Carcinoma cell implantation. In tumors derived from Lewis Lung Carcinoma cell-implanted ovariectomized, transgenic mice, the transcription and expression of hCG-driven, tumor-associated molecules remained elevated and enhanced animal mortality was observed. Cell-extrinsic βhCG can therefore induce pro-tumorigenic effects *in vivo* (even on tumor lineages not part of the reproductive axis), with ovarian products mediating an ameliorating influence.

## INTRODUCTION

Human chorionic gonadotropin (hCG), a glycoprotein hormone, is secreted by the pre-implantation embryo [1, 2]. It acts to “rescue” the corpus luteum, preventing its degeneration and enabling the sustained secretion of progesterone [3]. Interestingly, hCG (or its constituent alpha or beta subunits), is also secreted by trophoblastic cancers, as well as by various non-trophoblastic tumors such as of the breast, lung, liver, brain, prostate, head and neck, ovary, kidney, and of the neuro-endocrine system. In this regard, the fact that the processes of growth, inflammation, metastasis, apoptosis, angiogenesis and immune suppression are shared between the physiological state of pregnancy and the pathological state of cancer assumes relevance. Of additional interest are observations that in cancer patients the presence of hCG (or more often, the β subunit) is associated with poor prognosis in many instances [4–12].

On-going work has sought to establish the roles hCG might play in the promotion of tumorigenesis. For example, βhCG has been shown to stimulate the proliferation of bladder cancer cells *in vitro* [13]. hCG demonstrates angiogenic properties [14, 15], and induces the generation of VEGF, IL-8 and matrix metalloproteases (MMPs) in tumor cells; tumor invasiveness is enhanced, an effect neutralized by anti-hCG and anti-MMP antibodies. Secretion of versican also increases upon the addition of hCG to tumor cells; the proteoglycan then stimulates secretion of TNF-α and IL-6 (heightened levels of which are associated with metastasis, chemoresistance and poor patient prognosis [16, 17]) from immune cells in a TLR-2-dependent manner. Further, hCG induces the production of immunosuppressive cytokines which promote the differentiation of Treg cells [18]. hCG ameliorates drug-induced apoptosis of tumor cells [19, 20] and up-modulates multiple mediators of chemoresistance (including *Survivin, Hif-1α, Parp-1, Bcl-2, x-Flip, Klk10, Xiap, Ciap-1*); knock-down of many of these molecules abrogates hCG-mediated chemoresistance [20]. Targeting hCG, via either immunological or non-immunological means, has anti-tumor effects; anti-αhCG antibodies, as well as antisense oligonucleotides targeting αhCG, have an adverse influence on the growth of human lung tumor cells *in vitro* as well as *in vivo* [21]. Anti-sense oligonucleotides to βhCG, while effective on their own, further sensitize tumor cells to the anti-proliferative effects of c-Myc inhibition [22]. Stable transfection of an antisense βhCG oligonucleotide into JAr choriocarcinoma cells leads to suppression of βhCG protein synthesis and subsequently to an increase in apoptosis and diminished cell proliferation [23]. Anti-hCG immunization results in the inhibition of syngenic tumors in mice [18, 20] and similar vaccination in patients of colorectal cancer results in clinical benefit [24].

βhCG transgenic mice have been employed to further understanding of hCG-mediated tumorigenesis. Amongst other abnormalities, lactotrope pituitary adenomas are observed, resulting in an increase in serum prolactin and consequent infertility [25–27]. Prominent markers of tumorigenesis such as *Hmg2a, E2f1, Ccnd1, Prl, Gh, Gal, Pttg1 and Bmp4* are up-regulated, and Cdk inhibitors are down-regulated in the pituitary. Immunization against hCG effectively restores the expression of these genes to homeostatic levels and prevents the onset of infertility [25].

The availability of βhCG transgenic mice provides an opportunity to investigate the growth-promoting effects of βhCG (as an endogenous moiety) on responsive tumors of non-reproductive lineage *in vivo*. In the current study, transgenic and non-transgenic C57BL/6 × FVB^βhCG/−^ F1 female mice were characterized for the presence of βhCG and prolactin in the blood. The ovaries and pituitaries were histologically examined and transcript levels of hCG-driven, tumor-associated genes in the latter assessed. The effects of transgenically-expressed βhCG on the growth of implanted Lewis Lung Carcinoma cells (LLC1, murine lung tumor cells derived from C57BL/6 mice) were evaluated in terms of tumor volume and incidence. Additional analysis included the study of hCG-driven, tumor-associated transcripts in excised LLC1 tumors, as well as immuno-histochemical localization of their products. The individual effects of Cabergoline treatment as well as of ovariectomy on the growth of LLC1 tumors were determined.

## RESULTS

### Characterization of C57BL/6^−/−^ × FVB^βhCG/−^ F1 mice

**Figure 1:**
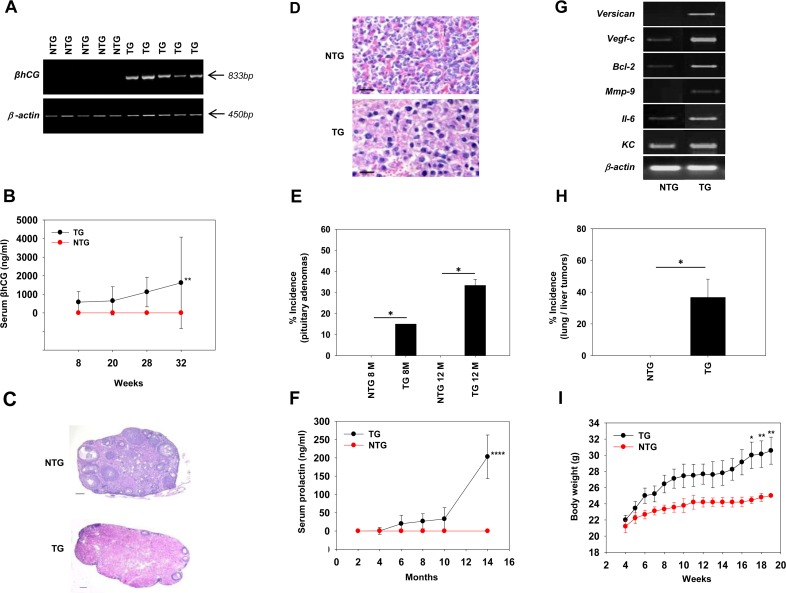
Characterization of C57BL/6^−/−^ × FVB^βhCG/−^ F1 mice **(A)** Genomic PCR for the βhCG transgene in NTG and TG mice. *β-actin* was employed as the house-keeping control. **(B)** Serum βhCG levels, as assessed by radioimmunoassay, as a function of age, in NTG (n = 8) and TG (n = 8) mice. Means ± SD are depicted. ^**^p<0.01 vs NTG mice by two-way ANOVA with 95% confidence interval. **(C)** Gross histology of ovaries derived from NTG and TG mice. Bars = 100 μm. **(D)** Histology of pituitaries derived from NTG and TG mice, demonstrating the presence of adenomas in the latter. Bars = 100 μm. **(E)** Incidence of pituitary adenomas in NTG (n = 8) and TG (n = 8) mice at 8 months [M] and 12 months [M]. ^*^p<0.02 by Mann-Whitney *U* test with 95% confidence interval. **(F)** Serum prolactin levels, as assessed by ELISA, as a function of age, in NTG (n = 8) and TG (n = 8) mice. Means ± SD are depicted. ^****^p<0.0001 vs NTG mice by two-way ANOVA with 95% confidence interval. **(G)** Reverse transcriptase-PCR for hCG-driven, tumor-promoting molecules on mRNA obtained from pituitaries derived from NTG and TG mice. *β-actin* was employed as the house-keeping control. **(H)** Incidence of spontaneously-arising liver and/or lung tumors in NTG (n = 12) and TG (n = 9) mice. ^*^p<0.02 by Mann-Whitney *U* test with 95% confidence interval. **(I)** Body weights of NTG (n = 9) and TG (n = 9) mice as a function of age. Means ± SD are depicted. ^*^p<0.02, ^**^p<0.01 vs NTG mice by two-way ANOVA with 95% confidence interval. NTG: Non-transgenic; TG: Transgenic.

Half of the female C57BL/6^−/−^ × FVB^βhCG/−^ F1 mice demonstrated the presence the βhCG transgene as expected (Figure 1A), since the transgene was heterozygously expressed in the male FVB parent. In transgenic mice, serum βhCG levels demonstrated an age-related increase (Figure 1B). While ovaries isolated from non-transgenic mice contained follicles at different stages of maturation, those from transgenic mice demonstrated evidence of hyper-luteinisation (Figure 1C). Pituitary adenomas were observed in transgenic mice (Figure 1D), the incidence of which demonstrated an age-related increase; such adenomas were not observed in non-transgenic mice (Figure 1D, 1E). Associated with the appearance of pituitary adenomas, serum prolactin levels rose as transgenic mice aged (Figure 1F). Transcripts of *Versican, Vegf-c*, *Bcl-2*, *Mmp-9*, *Il-6* and *KC* were up-modulated in pituitaries isolated from transgenic mice (Figure 1G); in *in vitro* studies, exogenous hCG has been shown to up-modulate transcription and expression of these molecules in cancer cells [18, 20]. Lung and liver tumors were observed in about a third of aging C57BL/6^−/−^ × FVB^βhCG/−^ F1 transgenic mice (Figure 1H). Transgenic expression of βhCG under the Ubiquitin C promoter has been associated with the appearance of metastatic mammary gland tumors in the liver and the lung [26]. While subsequent characterization of such tumors in the present model will reveal their true lineage, their existence is further indication of the association of βhCG with extra-gonadal tumorigenesis. An age-related increase in body weight occurred in transgenic mice (Figure 1I), an observation similar to multiple reports in a previously-described analogous model, and attributed to raised prolactin levels [25–27].

### Effect of transgenic βhCG expression on implanted LLC1 cells

#### Effects on growth

**Figure 2:**
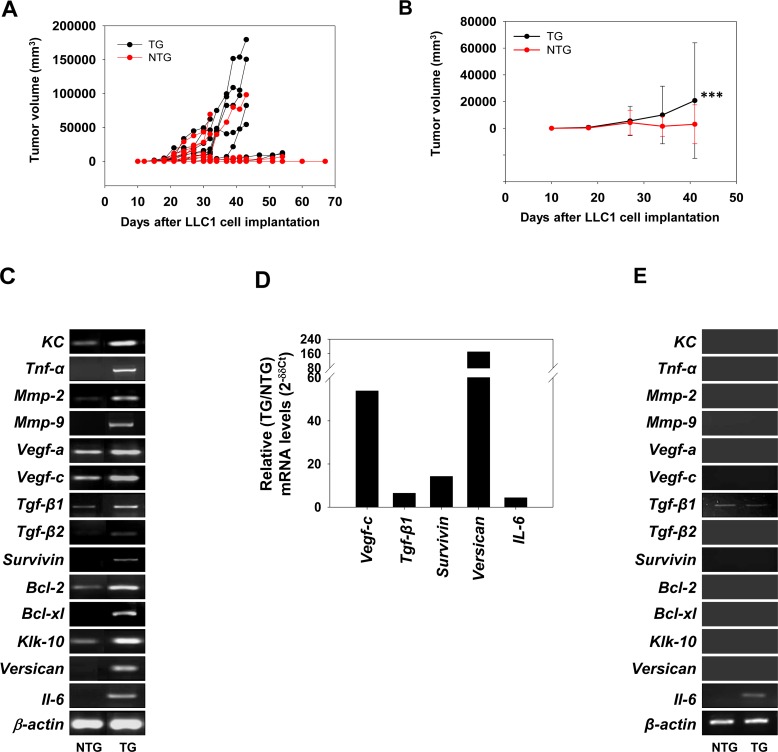
Effect of βhCG transgenesis on LLC1 tumor growth and on levels of hCG-driven, tumor-promoting molecules **(A)** Kinetics of LLC1 tumor growth in individual NTG (n = 28) and TG (n = 22) mice upon the subcutaneous implantation of LLC1 cells. **(B)** Average LLC1 tumor volumes in NTG (n = 28) and TG (n = 22) mice upon the subcutaneous implantation of LLC1 cells. Means ± SD are depicted. ^***^p<0.001 vs NTG mice by two-way ANOVA with 95% confidence interval. **(C)** Reverse transcriptase-PCR for hCG-driven, tumor-promoting molecules in LLC1 tumors derived from NTG and TG mice. *β-actin* was employed as the house-keeping control. **(D)** Real Time-PCR for hCG-driven, tumor-promoting molecules in LLC1 tumors. Data depicts fold-increase (TG/NTG) of mRNA after individual normalization against GAPDH mRNA. **(E)** Reverse transcriptase PCR for mRNA for hCG-driven, tumor-promoting molecules in spenocytes derived from NTG and TG mice. *β-actin* was employed as the house-keeping control. NTG: Non-transgenic; TG: Transgenic.

An enhancement in the kinetics of growth of LLC1 tumors was observed when LLC1 cells were subcutaneously implanted in transgenic mice versus when these cells were implanted in non-transgenic mice (Figure 2A); average tumor volumes (Figure 2B) as well as tumor incidence (Supplementary Figure 1) were increased. The presence of βhCG therefore appears to positively influence the growth of implanted lung tumor cells *in vivo*.

#### Effects on hCG-driven, tumor-associated molecules

Increased mRNA levels of several tumorigenesis-associated molecules known to be up-modulated by hCG in tumor cells [18, 20] were observed in LLC1 tumors isolated from transgenic mice, compared with LLC1 tumors isolated from non-transgenic mice. While representative data is depicted in Figure 2C, quantitative analysis (carried out using ImageJ software) is shown in Supplementary Figure 2. Real Time-PCR analysis lent general support to these findings (Figure 2D). Splenocytes derived from transgenic mice did not demonstrate such increases in mRNA levels (Figure 2E). These results indicate that, in transgenic mice, the up-modulation of tumor-promoting genes preferentially occurred in LLC1 tumor tissue.

### Effects of Caberoline administration on LLC1 tumors in transgenic and non-transgenic mice

**Figure 3:**
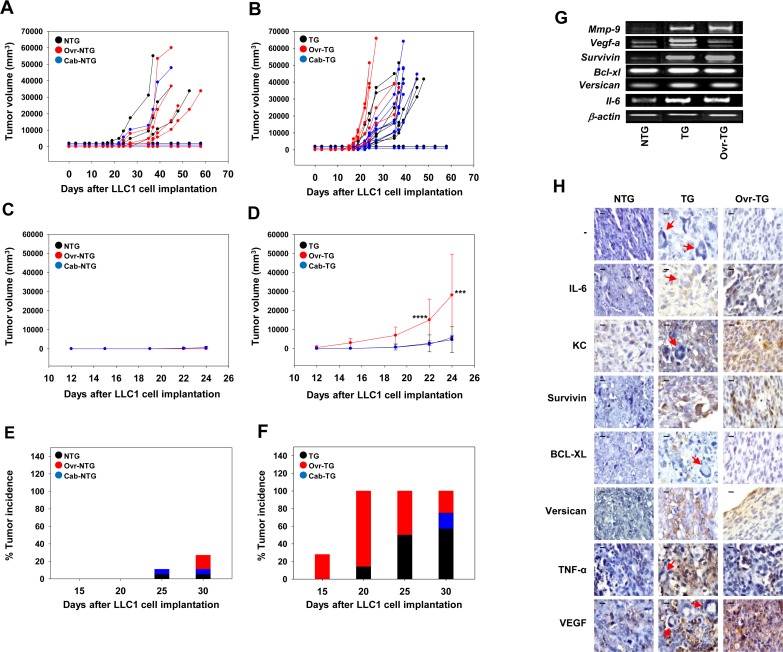
Effect of Cabergoline administration and ovariectomy on the development of LLC1 tumors in C57BL/6^−/−^ × FVB^βhCG/−^ F1 non-transgenic and transgenic mice **(A-D)** Kinetics of LLC1 tumor growth in LLC1 cell-implanted control, Cabergoline-administered (Cab) and ovariectomized (Ovr) NTG (A, C) and TG (B, D) mice. Data for individual mice (A, B) and group averages (C, D) is shown. In (C, D), Means ± SD are depicted. ^***^p<0.001, ^****^p<0.0001 vs control TG mice by two-way ANOVA with 95% confidence interval. **(E, F)** LLC1 tumor incidence in LLC1 cell-implanted control, Cabergoline-administered (Cab) and ovariectomized (Ovr) NTG (E) and TG (F) mice. Data in individual groups collated from [A] and [B] respectively. For A, C, E: NTG (n = 11); Ovr-NTG (n = 11); Cab-NTG (n = 9). For B, D, E: TG (n = 9); Ovr-TG (n = 7); Cab-TG (n = 9); **(G)** Reverse transcriptase-PCR for hCG-driven, tumor-promoting molecules on LLC1 tumors derived from NTG, TG and ovariectomized (Ovr) TG mice. **(H)** Immunohistochemical localization of IL6, KC, Survivin, BCL-XL, Versican, TNF-α and VEGF on LLC1 tumors derived from LLC1 cell-implanted NTG, TG and ovariectomized (Ovr) TG mice. ‘−‘ indicates the negative control. Red arrows in tumor sections derived from TG mice indicate giant tumor cells. Bars = 10 μm. NTG: Non-transgenic; TG: Transgenic.

Treatment of non-transgenic mice with Cabergoline (a potent dopamine receptor agonist) had minimal effects on mean LLC1 tumor volume, as well as on tumor incidence. Significantly, in transgenic mice as well, treatment with Cabergoline did not affect mean LLC1 tumor volume or incidence (Figure 3A-3F). Enhanced serum levels of prolactin were therefore not responsible for the increased incidence and volume of LLC1 tumors in transgenic mice.

### Ovariectomy

In order to assess the influence of ovarian products on the growth of implanted LLC1 cells, non-transgenic and transgenic mice were ovariectomized before cell implantation. To be able to rely on the outcomes of subsequent experimentation on these animals, it was important to first verify whether efficient surgical excision of the ovaries had been achieved. In non-transgenic mice, estrous cyclicity was employed as a measure; while intact non-transgenic mice displayed all four stages of the estrous cycle as expected, ovariectomized, non-transgenic mice were in a constant state of Diestrous (Supplementary Figure 3A). Estrous cyclicity could not be employed as a measure to assess the success of ovariectomy in transgenic mice, since intact transgenic mice themselves remained in a constant state of Diestrous, in consonance with previous reports in a analogous βhCG transgenic mouse strain [25, 27]; ovariectomy did not alter this state in transgenic mice, as expected (Supplementary Figure 3A). Instead, in transgenic mice, two additional read-outs known to be influenced by the ovaries (enhanced serum prolactin levels and increased body weight) were employed to assess the efficiency of ovariectomy; unlike in intact transgenic mice, serum prolactin levels remained low in ovariectomized transgenic mice (Supplementary Figure 3B) and increases in body weight were also not observed (Supplementary Figure 3C). These data indicate that surgical excision of the ovaries was efficacious in both non-transgenic and transgenic mice.

### Effects of ovariectomy on LLC1 tumors in transgenic and non-transgenic mice

#### Effects on tumor growth

LLC1 cells were implanted in control and ovariectomized, transgenic and non-transgenic mice. Ovariectomy in transgenic mice resulted in enhancement in LLC1 tumor volume, particularly at the early stages after cell implantation (Figure 3B, 3D). Coupled with this decrease in the lag phase in ovariectomized transgenic mice was an increase in LLC1 tumor incidence, with a hundred percent of ovariectomized transgenic mice exhibiting tumors twenty days after LLC1 cell implantation (Figure 3F). Neither a decreased lag phase nor an equivalent increase in LLC1 tumor incidence was observed in ovariectomized non-transgenic mice implanted with LLC1 cells (Figure 3A, 3C, 3E). This data was confirmed in three independent experiments, the data from which is depicted in Supplementary Figure 1 and can be summarized as follows: Tumor incidence in LLC1 cell-implanted ovariectomized transgenic mice was significantly higher compared with the tumor incidence in LLC1 cell-implanted intact transgenic mice. To a lesser extent, ovariectomy also enhanced tumor incidence in LLC1 cell-implanted non-transgenic mice compared with the tumor incidence in LLC1 cell-implanted intact non-transgenic mice. Finally, tumor incidence in LLC1 cell-implanted ovariectomized transgenic mice was significantly greater than tumor incidence in LLC1 cell-implanted ovariectomized non-transgenic mice. Absence of the ovaries therefore dramatically alters tumor progression in βhCG transgenic mice.

#### Effect on hCG-driven, tumor-associated molecules in transgenic and non-transgenic mice

Transcript levels of several hCG-driven, tumor-associated genes remained elevated in LLC1 tumors isolated from ovariectomized transgenic mice, compared to tumors isolated from non-transgenic mice (Figure 3G), an indication that enhanced levels, while dependent on the presence of βhCG, were not dependent upon the presence of the ovaries. Upon histological analysis, pleomorphic, giant tumor cells (GTCs) were observed in LLC1 tumors derived from transgenic mice (Figure 3H), the potential significance of which is discussed below.

Immunohistological analysis revealed that expression of several moieties including IL-6, KC, Survivin, BCL-XL, Versican, TNF-α and VEGF was enhanced in LLC1 tumors derived from transgenic mice compared with LLC1 tumors derived from non-transgenic mice. In LLC1 tumors derived from ovariectomized transgenic mice, levels of BCL-XL declined to undetectable levels. Staining for TNF-α also decreased on LLC1 tumors derived from ovariectomized transgenic mice (compared to LLC1 tumors derived from intact transgenic mice) but was still detectable, and higher than in LLC1 tumors derived from non-transgenic mice. In all other instances, levels of these factors remained elevated, or were enhanced, in LLC1 tumors derived from ovariectomized transgenic mice compared with LLC1 tumors derived from intact transgenic mice (Figure 3H). As with mRNA levels, these results also indicate that, for the most part, the enhancement in the expression of these moieties, while dependent on the presence of βhCG, was not dependent on the presence of the ovaries

#### Effect on mortality

**Figure 4:**
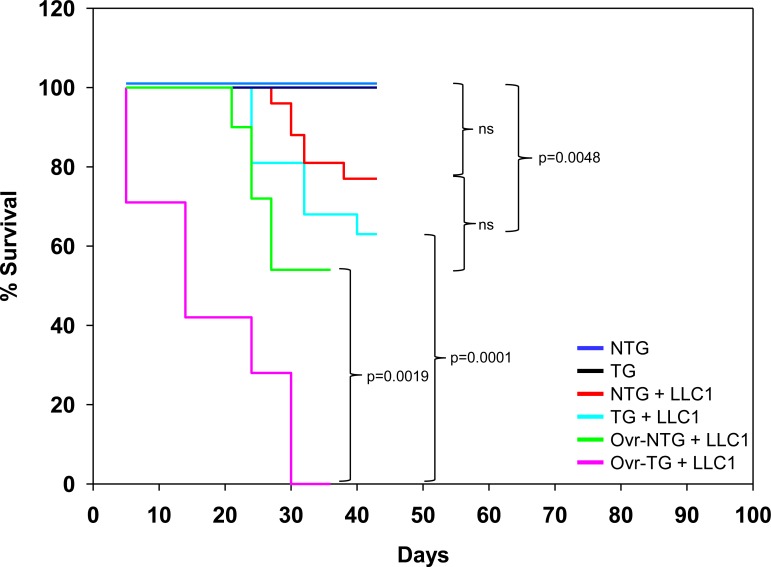
Kaplan-Meier survival analysis Mortality of NTG mice (n = 9), TG mice (n = 9) mice, LLC1-implanted NTG mice (n = 11), LLC1-implanted TG mice (n = 9), LLC1-implanted ovariectomized (Ovr) NTG mice (n = 11) and LLC1-implanted ovariectomized (Ovr) TG mice (n = 7). Statistical analysis was carried out using the Log Rank (Mantel-Cox) test. NTG: Non-transgenic; TG: Transgenic.

Neither non-transgenic mice nor βhCG transgenic exhibited mortality over the period of observation. While the implantation of LLC1 cells in non-transgenic mice did not result in significantly enhanced mortality over non-transgenic mice in which LLC1 cells had not been implanted, LLC1 cell implantation in transgenic mice did have a significant detrimental effect on mortality, compared with transgenic mice in which LLC1 cells had not been implanted. Further, while ovariectomy in non-transgenic mice did not significantly enhance mortality upon the implantation of LLC1 cells (compared with the mortality of intact, non-transgenic, LLC1 cell-implanted mice), the mortality of ovariectomized, LLC1-implanted, transgenic animals was significantly enhanced, compared with both intact, LLC1-implanted transgenic mice, as well as with ovariectomized, LLC1-implanted, non-transgenic mice (Figure 4). Taken together, these results indicate that the presence of βhCG, most significantly in the absence of the ovaries, has adverse effects on the life-span of mice bearing gonadotropin-responsive tumors.

## DISCUSSION

hCG is a hormone critical to the success of pregnancy, and its expression was originally believed to be restricted to that physiological state. Increasingly, the presence of hCG, or of its subunits, has been documented in cancers belonging to lineages which lie both within and outside the reproductive system. While interesting in itself, what makes such a finding additionally intriguing is the fact the presence of the βhCG subunit in cancer has been associated, quite extensively, with poor patient prognosis [5, 6, 9, 10–12]. While in experimental systems as well as in humans, anti-hCG immunization has been shown to have anti-tumor effects [18, 20, 24, 25, 28], the basis of the relationship between presence of the hormone and poor prognosis and has been inadequately elucidated, particularly *in vivo*. Some leads have emerged from *in vitro* experiments, employing either the whole hormone or the β subunit; beside enhancing tumor cell viability [18, 20] and inducing the proliferation of tumor cells [13], the hormone has been shown to promote the secretion of the mediators of angiogenesis and invasion, whilst also stimulating the secretion inflammatory molecules and immunosuppressive cytokines [18]. Such data gains significance in light of the fact that the physiological processes which characterize embryogenesis (for example, invasiveness, angiogenesis and immune suppression) overlap to a significant extent with the pathological processes which characterize carcinogenesis. Additionally, the hormone can induce a state of chemoresistance upon incubation with tumor cells [19, 20], an observation of considerable importance, since resistance to drug action is an important determinant of poor patient prognosis. In this regard, an interesting synergy has been observed between hCG and certain TLR ligands in the induction of chemoresistance, suggesting that the release of endogenous TLR ligands subsequent to drug-induced cell death may promote drug resistance in the presence of hCG [20]. Elucidation of the signalling events that bring about such outcomes could potentially result in new therapies, or the further rationalization of available therapeutic options.

The development of animal models in which βhCG acts as a “self” growth factor for tumors that lie outside the reproductive axis (much like it is increasingly believed to do in humans) represents a clear need; the current study is significant a step in that direction. Implantation of responsive tumor cells (towards which a competent immune system is tolerant) in βhCG transgenic mice allows elucidation of factors and processes that promote gonadotropin-promoted tumorigenesis *in vivo*. Transgenic C57BL/6 × FVB^βhCG/−^ F1 female mice displayed ovarian hyperplasia as well as pituitary tumors, in consonance with previous reports in an analogous system [25–27]. mRNA levels of several hCG-driven, tumor-promoting genes (as identified in previous work [18, 20]) were up-modulated in pituitary adenomas. Implantation of LLC1 cells in transgenic mice resulted in enhanced tumor growth, as well as heightened mRNA levels of hCG-driven, tumor-promoting genes in such tumors. Levels of such mRNA in splenocytes derived from transgenic mice remained unaltered, arguing against a global up-modulation of hCG-driven, tumor-associated molecules, and a tumor-specific effect. The fact that LLC1 tumors derived from transgenic mice exhibited the presence of GTCs assumes significance in light of the fact that βhCG-secreting cancers such as germinomas, as well as bone and lung carcinomas, are characterized by the presence of such GTCs [29–32]; such a tumor phenotype has been frequently associated with increased aggressiveness and poor patient prognosis.

Ovariectomy in transgenic (but not in non-transgenic) mice dramatically altered the kinetics of LLC1 tumor growth; increased tumor incidence and a decreased lag phase were associated with increased tumor volumes, with enhanced mortality as a consequence. These results strongly suggest that the pro-tumorigenic effects of cell-extrinsic βhCG observed upon implantation of LLC1 tumor cells in transgenic mice are not a consequence of indirect effects elicited upon ovarian stimulation; they contrarily imply that ovarian products act to ameliorate βhCG-driven tumorigenesis *in vivo*. The identity of the ovarian product(s) that influence the growth of tumor cells in a gonadotropin-containing milieu *in vivo* is the focus of current investigation; the fact progesterone [33–37] as well as estradiol [38, 39] demonstrate anti-tumor effects is of obvious relevance. In this context, since an association exists between post-menopausal status (particularly in women who experience menopause after 55 years of age) and the increased risk of cancers of different lineages [40, 41], whether circulating hCG (low levels of which have been detected after menopause [42]) can be an aggravating factor would be of interest to evaluate. Further, whether additional primary tumors arise with greater frequency at distal sites, in the presence of an initial primary βhCG-secreting tumor, deserves investigation. Were such associations to exist, the case for anti-hCG vaccination would be further enhanced, given reports of the anti-tumor efficacy of such intervention in several experimental systems.

## MATERIALS AND METHODS

### Ethics statement

Investigations have been conducted in accordance with the ethical standards and according to the Declaration of Helsinki and according to national and international guidelines and have been approved by the Institutional Animal Ethics Committee of the National Institute of Immunology (IAEC Numbers: 231/10, 392/15). All animal experiments carried out in accordance with guidelines laid down by the Committee for the Purpose of Control and Supervision of Experiments on Animals of the Government of India.

### Cell culture

LLC1 (murine Lewis Lung Carcinoma) cells were obtained from the American Type Culture Collection (ATCC) and were used within 3 months of receipt. DMEM (GIBCO, Invitrogen) was supplemented with 10% foetal bovine serum (Biological Industries) and an antibiotic-antimycotic cocktail (GIBCO, Invitrogen).

### Generation of C57BL/6 × FVB^βhCG/−^ F1 mice

Six week-old inbred C57BL/6 female mice, obtained from the Small Animal Facility of the National Institute of Immunology, were crossed with FVB^βhCG/−^ male mice; these mice express βhCG under the Ubiquitin promoter [26]. Pups in the F1 generation were weaned at 21 days.

### Genomic PCR for βhCG

Peripheral blood was collected via the retro-orbital vein under anaesthesia. Genomic DNA was isolated using a kit (Genetix). Transgenic and non-transgenic animals were distinguished based on the presence or absence of the βhCG transgene as assessed by PCR; primer sequences for βhCG and *β-actin* are listed in Supplementary Table 1. The following protocol was followed: Denaturation at 95°C, annealing at 55°C (for βhCG) or 60°C (for *β-actin*), extension at 72°C for 30 cycles.

### Serum βhCG

Serum βhCG was quantified by radio-immunoassay. A murine monoclonal antibody specific for βhCG was employed for this purpose. Briefly, diluted sera were incubated with the antibody, 4% normal horse serum and ^125^I-hCG (40 μCi/μg, ≅ 10,000 cpm) at 4°C for 16 hrs. Polyethylene glycol (Mw 6000; Sigma) at a final concentration of 12.5% was then added. Centrifugation was carried out at 1840 g at 6°C to precipitate immune complexes. Supernatants were decanted and radioactivity in the pellet determined in a gamma counter. Pure hCG (1.25 ng/ml – 40 ng/ml) was employed as standard and serum βhCG was quantified by comparisons with the standard curve obtained upon linear regression.

### Serum prolactin

An ELISA kit (R&D Systems) was employed for the quantification of serum prolactin. Optical densities were measured at 450 nm on an ELISA reader (BioTeK μquant). Pure prolactin (0.0119 ng/ml – 20 ng/ml) was employed as standard and serum prolactin was quantified by comparisons with the standard curve using Gene5 software.

### Estrous cyclicity

The vagina was flushed with normal saline and smears examined under a light microscope. Stages of the estrous cycle were enumerated as Proestrous (a few nucleated cells and an absence of leucocytes), Estrous (a large number of cornified cells), Meta-estrous (a few cornified cell and a small number of leucocytes) or Diestrous (leucocytes and a few nucleated cells).

### LLC1 cell implantation

Six week-old female transgenic and non-transgenic C57BL/6 × FVB^βhCG/−^ F1 mice were subcutaneously implanted with 40,000 LLC1 cells. Tumor volumes were measured at regular intervals employing the formula 4/3πr^3^ (l: length; w: width, using the largest axes of the tumor; r= (l+w)/2). Tumors were isolated and preserved for RNA isolation as well as fixed in 10% formaldehyde and processed for immuno-histochemistry.

### Cabergoline administration

Five week-old transgenic and non-transgenic C57BL/6 × FVB^βhCG/−^ F1 mice were treated with Cabergoline (a dopaminergic receptor agonist which inhibits the secretion of prolactin [43]) to assess the effects of the hormone on the growth on implanted LLC1 cells. Cabergoline (500 μg/kg; Sigma) was intra-peritoneally injected three times over the course of one week, in accordance with a regimen previously described for βhCG transgenic mice [27]; 0.25% methyl cellulose (Sigma) was employed as the vehicle. LLC1 cells were implanted at Week 6.

### Ovariectomy

Five week-old transgenic and non-transgenic C57BL/6 × FVB^βhCG/−^ F1 mice were ovariectomized to assess the effects of ovarian products on the growth on implanted LLC1 cells. Ovaries were surgically removed under local anaesthesia using Ketamine (Themis medicare)-Xylazine (Indian Immunologicals). Silk sutures were administered using a half-circle needle (No. 20). Betadine (Win medicare) and Neosporin (GlaxoSmithKline) were topically applied. Enrofloxacin (0.1 mg/ml) and ibuprofen (0.25 mg/ml) were added to the drinking water. After a period of recovery, LLC1 cells were implanted at Week 9.

### Reverse Transcriptase PCR (RT-PCR), Real Time PCR

Total RNA from tumor tissue was isolated using TRiZOL or by using a kit (Qiagen). RT-PCR was performed for interleukin-8 (*KC*), tumor necrosis factor-α (*Tnf-α*), matrix metallo-proteinase-2 (*Mmp-2*), matrix-metallo proteinase-9 (*Mmp-9*), vascular endothelial growth factor-a (*Vegf-a*), vascular endothelial growth factor-c (*Vegf-c*), tumor growth factor-β (*Tgf-β1*), tumor growth factor-β (*Tgf-β2*), survivin, B cell lymphoma-2 (*Bcl-2*), B cell lymphoma extra-large (*Bcl-xl*), kallikrien-like kinase 10 (*Klk- 10*), versican, interleukin (*Il-6*), interleukin-10 (*Il-10*), cellular FLICE inhibitor protein (*c-flip*) and X-linked inhibitor of protein (*Xiap*), using Taq DNA polymerase (Biotools)*. β-actin* was employed as the house-keeping control. Primers sequences are listed in Supplementary Table 2.

Real time PCR was performed employing Syber green master mix (Mesa green, 2x PCR master mix for SYBR green I; Eurogenetec, Applied Biosystems); primer sequences are listed in Supplementary Table 3.

### Immunohistochemistry

3 μm sections were cut from paraffin-embedded tumor tissue. After de-paraffinization, antigen retrieval was carried out by incubating sections in Tris EDTA buffer for 20 mins at 95°C. Endogenous peroxidase activity was blocked by incubation in “Peroxidase Block” (Abcam) for 10 mins. Sections were washed and then incubated in “Protein Block” (Abcam) for 20 mins. Antibodies against IL-6, KC, Survivin, BCL-XL, Versican, TNF-α and VEGF (Abcam) were incubated on individual slides at 4°C for 16 hrs in a moist chamber. An ABC (Avidin Biotin conjugate) kit (Abcam) was employed to visualize reactivity. Images (40X) were captured on a Leica microscope using a Leica ICC50W camera and processed using LAS V4-10 software.

### Statistical analysis

Statistical analysis was carried out using two-way ANOVA, the Student's *t* test or the Man-Whitney *U* test, as appropriate. The influence of LLC1 cell implantation on animal survival was assessed by Kaplan-Meier analysis, using the Log Rank (Mantel-Cox) test.

## SUPPLEMENTARY MATERIALS FIGURES AND TABLES



## Abbreviations

LLC1: Lewis Lung Carcinoma
hCG: Human chorionic gonadotropin
