# Supplementary material for: The transgenic expression of the β-subunit of human chorionic gonadotropin influences the growth of implanted tumor cells
Source: Oncotarget. 2018 Oct 5;9(78):34670–80. doi: 10.18632/oncotarget.26158 (PMC6205172; doi:10.18632/oncotarget.26158)
Supplement: Supplementary file 1 [file oncotarget-09-34670-s001.pdf]

The transgenic expression of the β-subunit of human chorionic gonadotropin influences the growth of implanted tumor cells

SUPPLEMENTARY MATERIALS

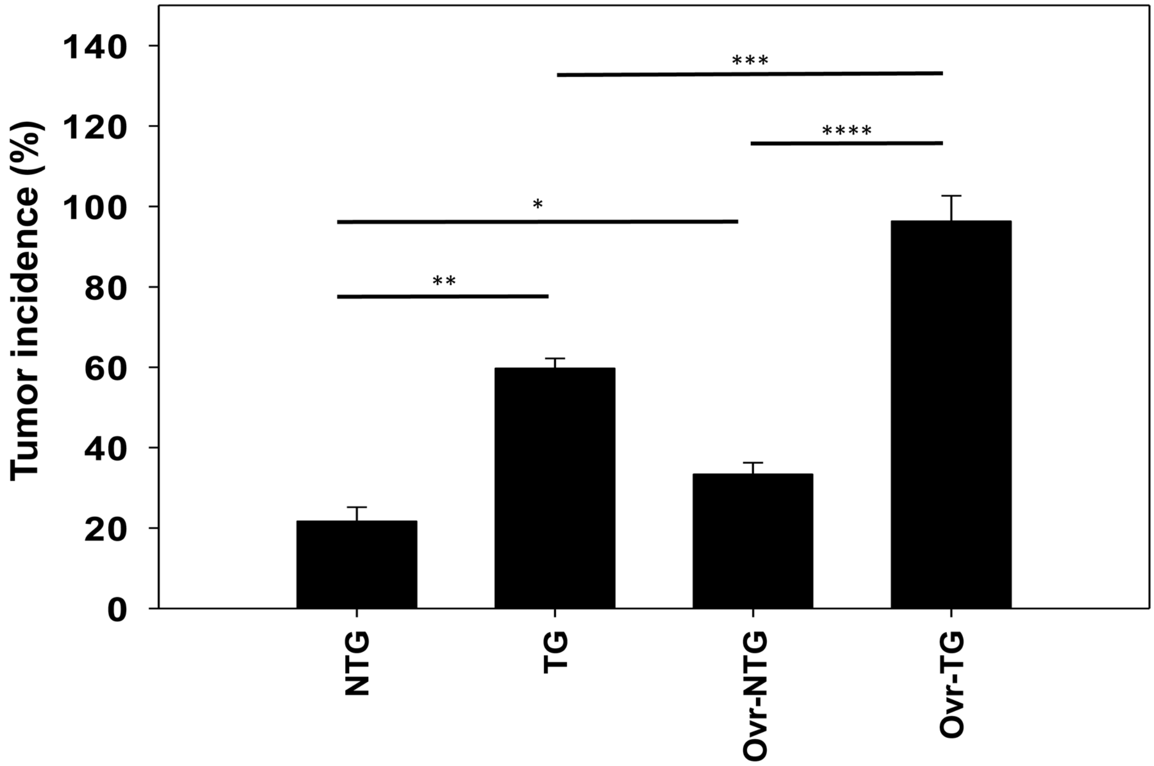

Supplementary Figure 1: Incidence of LLC1 tumors in C57BL/6$^{-/-}$ × FVB$^{βhCG/-}$ F1 non-transgenic, transgenic, ovariectomized non-transgenic and ovariectomized transgenic mice. Cumulative data (at Day 30, after LLC1 cell implantation from three independent experiments) of tumor incidence in NTG (n = 48), TG (n = 40), ovariectomized (Ovr) NTG (n = 28) and ovariectomized (Ovr) TG (n = 22) mice upon the subcutaneous implantation of LLC1 cells. Means ± SD are depicted. $^{*}p<0.02$, $^{**}p<0.01$, $^{***}p<0.001$, $^{****}p<0.0001$ by one-way Mann-Whitney *U* test with 95% confidence interval.

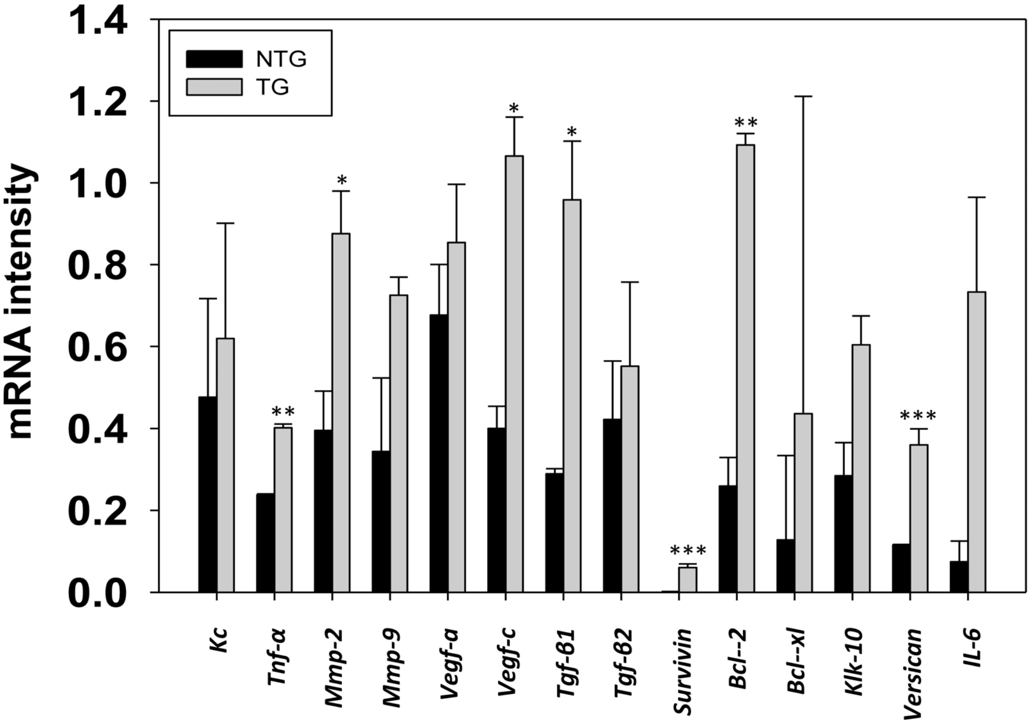

Supplementary Figure 2: Quantification of expression of hCG-driven tumor-promoting molecules. RT-PCR for hCG-driven, tumor-promoting molecules in LLC1 tumors derived from NTG (n = 12) and TG (n = 12) mice. Data depicts mean intensity (by ImageJ analysis) of PCR bands; intensities were normalized against *β-actin*. Means ± SD are depicted. $^{*}p<0.05$ $^{**}p<0.01$, $^{***}p<0.001$ by Mann-Whitney *U* test with 95% confidence interval. NTG: Non-transgenic; TG: Transgenic.

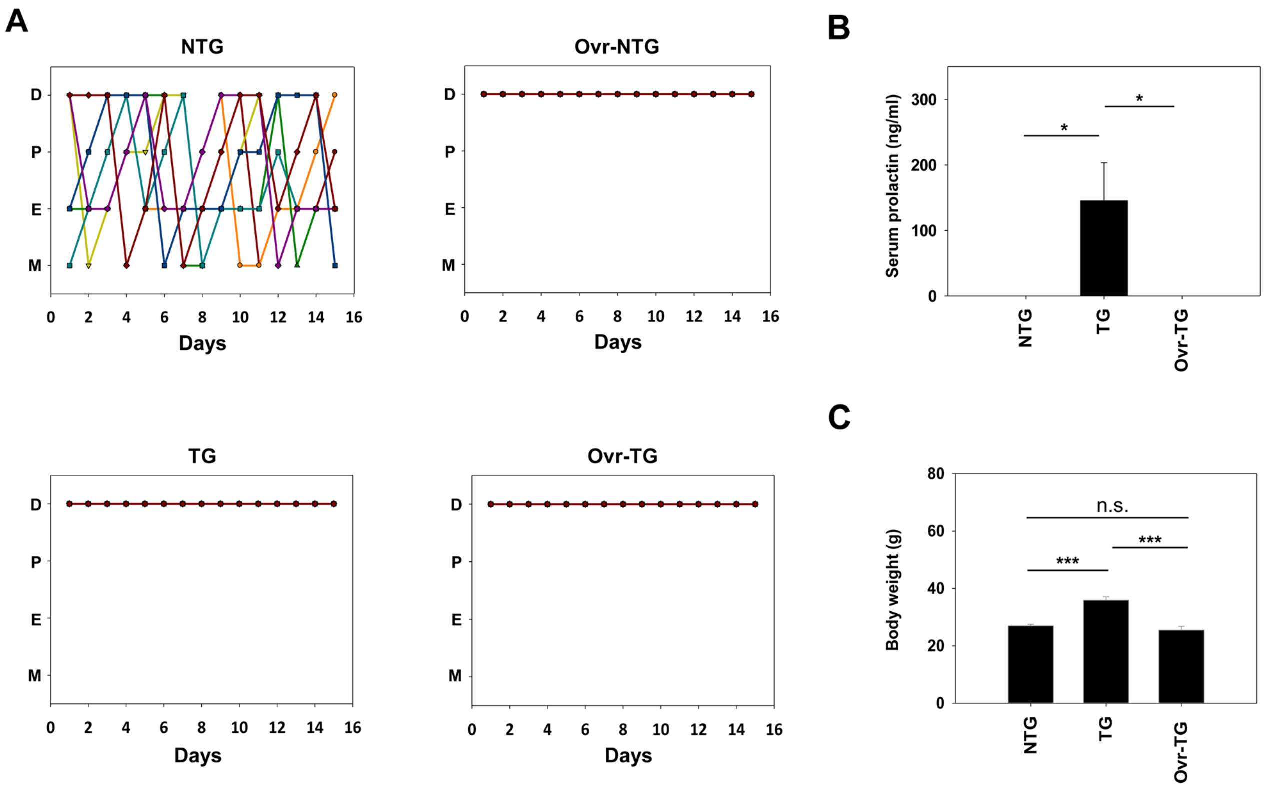

Supplementary Figure 3: Characterization of ovariectomized C57BL/6$^{-/-}$ × FVB$^{\beta hCG/-}$ F1 mice. **(A)** Estrous cyclicity in NTG (n = 9), ovaiectomized (Ovr) NTG (n = 9), TG (n = 9) and ovariectomized (Ovr) TG (n = 7) mice. Colours depict individual mice. D: Diestrous; P: Proestrous; M: Metaestrous; E: Estrous. **(B)** Serum prolactin levels (at 14 months) in NTG (n = 7), TG (n = 8) and ovariectomized (Ovr) TG (n = 7) mice. Means ± SD are depicted. *p<0.02 by one-way Student's *t*-test. **(C)** Body weights (at 14 months) in NTG (n = 7), TG (n = 6) and ovariectomized (Ovr) TG (n = 5) mice. Means ± SD are depicted. ***p<0.001 by one-way Student's *t*-test. n.s.: Not significant. NTG: Non-transgenic; TG: Transgenic.

Supplementary Table 1 : Primers and annealing temperatures for genomic PCR

Gene	Forward Primer (5'-3')	Reverse Primer (5'-3')	Annealing Temp (°C)
βhCG	CGCGCCCTCGTCGTGTC	AAGCGGGGGTCATCAGGTC	55
β-actin	AACGCAGCTCAGTAACAGTC	ATCCGTAAAGACCTCTATGC	60

Supplementary Table 2: Primers and annealing temperatures for Reverse Transcriptase-PCR

Gene	Forward Primer (5'-3')	Reverse Primer (5'-3')	Annealing Temp (°C)
KC	ACCCAAACCGAAGTCATAGCC	TGGGGACACCTTTTAGCATC	60
Tnf-α	CCTGCCCCAAGGACACCCCT	CTCCAGGACACCCCGGCCTT	60
Mmp-2	CACCTACACCAAGAACTTCC	AACACAGCCTTCTCCTCCTG	58
Mmp-9	TTGAGTCCGGCAGACAATCC	CCTTATTCCACGCGAATGACG	58
Vegf-a	CTGTGCAGGCTGCTGTAACG	GTTCCCGAAACCCTGAGGAG	58
Vegf-c	TGCTGAGGTAACCTGTGCTG	CAAGGCTTTTGAAGGCAAAG	60
Tgf-β1	TCGATCGCTACCCGGCGTTC	TAGATGGCGTTGTTGCGGTCC-	65
Tgf-β2	TCGTCCCTTTTGGCCGGAGGAT	TGGGCGGGATGGCATTTTCG	60
Survivin	CCACCGCATCTCTACATTCA	TATGTTCCTCTATGGGGTCG	55
Bcl-2	GCGCAAGCCGGGAGAACA	AGACGTCCTGGCAGCCAT	65
Bcl-xl	CATCCAAACTGCTGCTGCTGTGG	TTATCTTGGCTTTGGCATCCTG	50
Klk-10	GATCACCTGCTGCTTCTTC	CACTCTGGCAAGGGTCCTG	60
Versican	AGCGGCAAGTTCAGAGTGT	GCCCAGAACGGAAATATCAA	60
Il-6	TGGTGCTGACCTCTGGACGCTT	TGTGGCGTGTTCTGGGCTGT	60
Il-10	ACTGTGACTTTGGGGACCTG	TTCCCAAGGAAGAACCCCTCCCA	60
c-Flip	GAGGTTGAGGGACTTGGCATG	TCAGCAGGACCCTATAATCAG	60
Xiap	GAAGACCCTTGGGGAACAACA	GTCCTTGAAACTGAACCCCA	60

Supplementary Table 3: Primers for Real Time-PCR

Gene	Forward Primer (5'-3')	Reverse Primer (5'-3')
Vegf-c	AGCCCACCCTCAATACCAG	GCTGCTCCAAACTCCTTCC
Tgf- β1	CGCCATCTATGAGAAAACC	GTAACGCCAGGAATTGT
Survivin	TCATCCACTGCCCTACCGAGAAC	TCTATCGGGTTGTCATCGGGTTC
Versican	TCCTGATTGGCATTAGTGAAG	CTGGTCTCCGCTGTATCC
Il-6	CGATGATGCACTTGCAGAAA	TGGAAATTGGGGTAGGAAGG
β-Actin	CTCTGGCTCCTAGCACCATGAAGA	GTAAAACGCAGCTCAGTAACAGTCCG
18s rRNA	CGAAAGCATTTGCCAAGAAT	AGTCGGCATCGTTTATGGTC
